# Comparison of data processing strategies using commercial *vs*. open-source software in GC-Orbitrap-HRMS untargeted metabolomics analysis for food authentication: thyme geographical differentiation and marker identification as a case study

**DOI:** 10.1007/s00216-024-05347-0

**Published:** 2024-05-28

**Authors:** Araceli Rivera-Pérez, Antonia Garrido Frenich

**Affiliations:** Research Group “Analytical Chemistry of Contaminants”, Department of Chemistry and Physics, Research Centre for Mediterranean Intensive Agrosystems and Agrifood Biotechnology (CIAIMBITAL), Agrifood Campus of International Excellence (ceiA3), University of Almeria, 04120 Almeria, Spain

**Keywords:** High-resolution mass spectrometry, Gas chromatography, Data analysis, MS-DIAL, Compound Discoverer, Metabolomics

## Abstract

Untargeted analysis of gas chromatography–high-resolution mass spectrometry (GC-HRMS) data is a key and time-consuming challenge for identifying metabolite markers in food authentication applications. Few studies have been performed to evaluate the capability of untargeted data processing tools for feature extraction, metabolite annotation, and marker selection from untargeted GC-HRMS data since most of them are focused on liquid chromatography (LC) analysis. In this framework, this study provides a comprehensive evaluation of data analysis tools for GC-Orbitrap-HRMS plant metabolomics data, including the open-source MS-DIAL software and commercial Compound Discoverer™ software (designed for Orbitrap data processing), applied for the geographical discrimination and search for thyme markers (Spanish *vs*. Polish differentiation) as the case study. Both approaches showed that the feature detection process is highly affected by unknown metabolites (Levels 4–5 of identification confidence), background signals, and duplicate features that must be carefully assessed before further multivariate data analysis for reliable putative identification of markers. As a result, Compound Discoverer™ and MS-DIAL putatively annotated 52 and 115 compounds at Level 2, respectively. Further multivariate data analysis allowed the identification of differential compounds, showing that the putative identification of markers, especially in challenging untargeted analysis, heavily depends on the data processing parameters, including available databases used during compound annotation. Overall, this method comparison pointed out both approaches as good options for untargeted analysis of GC-Orbitrap-HRMS data, and it is presented as a useful guide for users to implement these data processing approaches in food authenticity applications depending on their availability.

**Graphical Abstract:**

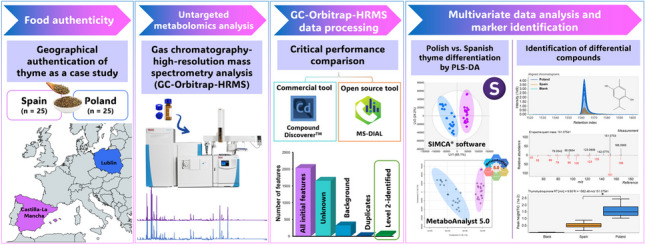

**Supplementary Information:**

The online version contains supplementary material available at 10.1007/s00216-024-05347-0.

## Introduction

Nowadays, there is increasing interest in moving toward the consumption of natural food products with beneficial properties such as herbs and spices [1]. Particularly, the current demand for spices and herbs is growing in Europe due to interest in new flavors, more healthy dietary habits, and the rise of the so-called ethnic gastronomy in Western civilizations. According to the Observatory of Economic Complexity (OEC), the global herb and spice trade has increased by 82.3% in the last decade (from 1.98 to 3.61 billion dollars between 2010 and 2020), with China and India as the main exporters [2, 3]. Nevertheless, herbs and spices are vulnerable to food fraud for a high number of reasons, including complex and long supply chain systems, high demand and limited production, and the fact that they are mostly supplied as ground powder, increasing their fraud vulnerability [4, 5]. This fact has led to the continuous demand for analytical methods to detect potential fraud practices.

The chemical composition of plant-derived food products is directly linked to their quality, being an indicator of the product's genuineness, including their origin, quality, processing, and authenticity (or adulteration) characteristics [6]. The metabolomics composition is highly dependent on diverse factors (e.g., growing conditions, production system, post-harvest processing such as drying or milling processes, or storage conditions), leading to variations in metabolite levels [7]. For these reasons, assessing alterations in the phytochemical composition of plant-derived foodstuffs through metabolomics approaches is one of the strategies of choice to monitor food authenticity [6].

Recently, metabalomics approaches based on chromatography coupled to high-resolution mass spectrometry (HRMS) have gained attention as one of the most reliable analytical tools for food authenticity assessment of complex matrices, such as herbs and spices, including geographical discrimination [8, 9], quality control [10], and adulteration [11] studies, being most of them based on ultra-high performance liquid chromatography (UHPLC)-HRMS analysis. A remarkable strength of HRMS is that accurate mass acquisition (by Orbitrap and time-of-flight, TOF, analyzers) greatly reduces the number of candidates per detected metabolite leading to a highly confident compound identification in metabolomics studies [12]. In contrast, up to now, only few gas chromatography (GC)-HRMS-based metabolomics applications are described in the herb and spice authentication field, including HRMS (Orbitrap)-based studies for geographical differentiation and processing traceability of black pepper [13] and thyme [14] by volatile fingerprinting.

As a result of the high number of detected metabolites (features) arising from raw GC-HRMS acquisition, automatic data analysis tools are necessary to manage untargeted omics data. In the field of HRMS-based metabolomics, there are different data processing options, including open-source tools such as MS-DIAL [15] and commercial packages such as Compound Discoverer™ (the most widely used data analysis tool for Thermo Fisher Scientific instruments). The capabilities of data processing tools may be compared depending on the tested approach, i.e., targeted or untargeted data analysis. The first one is usually focused on the analysis of a reference standard mixture where the method performance is assessed through the detection, identification, and quantification of expected features, being limited to these targeted compounds that often can not reflect complex situations found in the routine analysis [16]. On the other side, data processing performance is investigated in untargeted data analysis approaches by considering real samples. It constitutes a great challenge compared with targeted strategies, especially considering the limited knowledge of the features present in the samples due to the untargeted nature of the analysis, and that compound identification is greatly dependent on the availability of well-updated databases used for feature annotation, leading to considerable different detection and annotation findings from the same dataset [16, 17]. In this context, few studies have compared the performance of available data analysis tools for HRMS data processing. For instance, Li et al. [17] evaluated the performance of five software (open-source ones, namely MS-DIAL, MZmine 2, and XCMS; and commercial MarkerView and Compound Discoverer™ software) by considering a benchmark sample set consisting of 1100 compounds (drugs and metabolites) acquired using two advanced HRMS platforms (TOF and Q-Orbitrap analyzers). In another study, six data processing tools (including MS-DIAL and Compound Discoverer™, among others) were comprehensively evaluated for UHPLC-Q-Orbitrap-HRMS targeted and untargeted plant metabolomics analysis (licorice, tea, and tobacco), revealing that the feature extraction performance may vary in untargeted analysis of real samples, and as a result, only a few detected features were common within the tested processing methods for the same dataset [16]. Similarly, open-source MS-DIAL and patRoon platforms were used for the UHPLC-Q-Orbitrap-HRMS untargeted analysis of biological samples (plasma and feces) to discover biomarkers from the Parkinson’s disease metabolome [18]. As a result of the current trend towards “open science” workflows, some open-source tools (e.g., MS-DIAL) are gaining special attention to manage UHPLC-HRMS data, including different applications such as untargeted analysis for water contamination assessment [19], phytochemical profiling of herbal medicines [20], and metabolomics approaches to reveal the post-harvest processing influence on the chemical composition of plant matrices [21]. Notably, most previous studies were focused on UHPLC-HRMS data processing, and only a few applications of the MS-DIAL software for GC–MS data analysis have been reported in the literature, all of them dealing with untargeted low-resolution mass spectrometry (LRMS) information [22, 23]. Bearing in mind that limited attention had been paid to GC-HRMS data processing options, this study not only provides for the first time the application of an open-source data processing workflow based on the MS-DIAL software to manage GC-Orbitrap-HRMS untargeted plant metabolomics data, but also a comprehensive performance comparison with the commercial Compound Discoverer™ software, the tool of choice for Thermo Fisher Scientific instruments’ users. Both untargeted approaches were applied for the geographical differentiation (Spain and Poland) of thyme samples as the case study to notice how data processing strategies may influence the metabolome overview and the identification of differential compounds (markers) highlighted by further multivariate data analysis tools. Thus, this study offers guidance on MS-DIAL and Compound Discoverer™ suitable processing parameters for users who worked in GC-HRMS-based plant metabolomics for food authenticity and traceability applications.

## Materials and methods

### Chemicals

GC–MS grade ethyl acetate (AcOEt) of purity ≥ 99.5% was supplied by Honeywell Riedel-de-Haën (Seelze, Germany). Kovats retention indices (KI) were estimated considering a certified reference material of C_7_-C_40_ saturated alkanes (1000 μg/mL of each compound in *n*-hexane) obtained from Sigma-Aldrich (St. Louis, MO, USA).

### Samples of the study and sample pretreatment

Thyme samples were supplied by Sabater Spices (Murcia, Spain). Two different geographical origins were assessed as the case study: Poland (Lublin, *n* = 25) and Spain (Castilla-La Mancha, *n* = 25). Polish thyme samples were collected in August 2020 and Spanish ones were in July 2020. Before sample extraction and analysis, thyme samples were ground (final particle size of 0.2 mm) using an ultra centrifugal mill (ZM200, Retsch GmbH, Germany) for 10 min at 8000 rpm (3584 rcf, relative centrifugal force). All the samples were stored in airtight packaging at room temperature until further GC-Orbitrap-HRMS analysis.

### Sample preparation for GC-Orbitrap-HRMS metabolomics analysis

Thyme samples were extracted as described in previous research using simple and effective ultrasound-assisted extraction (UAE) [14]. Briefly, 200.00 ± 0.01 mg of sample was weighed in a 15 mL-polypropylene tube using a research-grade analytical balance (Ohaus^®^, Nänikon, Switzerland), 4 mL of AcOEt was added, and the samples were introduced in an Elmasonic S 80 H ultrasonic bath (Elma Schmidbauer, Germany) for 30 min (at 37 kHz) at room temperature. Then, the sample extracts were centrifuged (at 5500 rpm or 4400 × *g* for 10 min) using a Frontier™ 5816 centrifuge (Ohaus^®^, Nänikon, Switzerland) and filtered through 0.45 µm nylon filters. Thyme extracts were stored at − 21 °C until further analysis. All the samples were randomly extracted and analyzed to avoid analysis bias. Procedure blanks were also prepared for the instrument clean-up and to remove potential background signals during further data processing.

### GC-Orbitrap-HRMS instrumental analysis

GC-Orbitrap-HRMS metabolomics analysis of thyme extracts was carried out using a Trace 1310 GC chromatograph equipped with a TriPlus RSH autosampler coupled to a Q-Exactive Orbitrap mass analyzer (Thermo Fisher Scientific, Waltham, MA). Metabolite separation was performed using a BP5MS capillary column (30 mm × 0.25 i.d., 0.25 µm particle size) from SGE Analytical Science (Victoria, Australia). The GC oven temperature was programmed as follows: initial oven temperature of 60 °C (held for 2 min), increased from 60 to 180 °C (at 20 °C/min) and increased to 310 °C (at 10 °C/min, held for 10 min). The total running time was 31 min. Helium (99.999%) was used as the carrier gas and its flow rate was 1 mL/min. The injector temperature was set at 250 °C and the volume injection was 1 µL using the splitless mode (splitless time of 3 min). HRMS data acquisition was performed using the full scan-MS mode (profile mode) with electron ionization (EI at 70 eV) within the *m/z* range of 50–500 (scan time of 200 ms) at a resolution power of 60,000 full width at half maximum (FWHM) at *m/z* 200.

### Data processing strategies tested for GC-Orbitrap-HRMS metabolomics data

#### Dataset under study: Polish vs. Spanish thyme geographical differentiation

As previously described, the dataset consisted of a total of 50 thyme GC-Orbitrap-HRMS raw files, split into 25 Polish thyme samples and 25 Spanish ones. Additionally, a total of 6 GC-Orbitrap-HRMS raw files corresponding to procedure blanks analyzed at the beginning, every 10 analyzed samples, as well as at the end of the analysis batch, were considered during data processing to remove background signals. This dataset was specifically designed to evaluate the performance of data processing software in plant untargeted metabolomics analysis for applications focused on food authentication and the corresponding search for metabolite markers.

#### Data processing workflow using commercial Compound Discoverer™ software

Compound Discoverer™ software allows untargeted data processing of GC/LC-HRMS raw data specifically acquired from Thermo Fisher Scientific instruments. The software streamlines compound identification in metabolomics applications since it provides pre-defined workflow templates, as well as the possibility of flexible and fully customizable data processing workflows designed by the users. A further advantage of Compound Discoverer™ software for metabolomics applications is that the platform includes univariate data analysis to notice key compounds between sample groups (e.g., two-sample *t*-tests, fold change (FC) analysis). In this study, Compound Discoverer™ software version 3.3 was used.

**Fig. 1:**
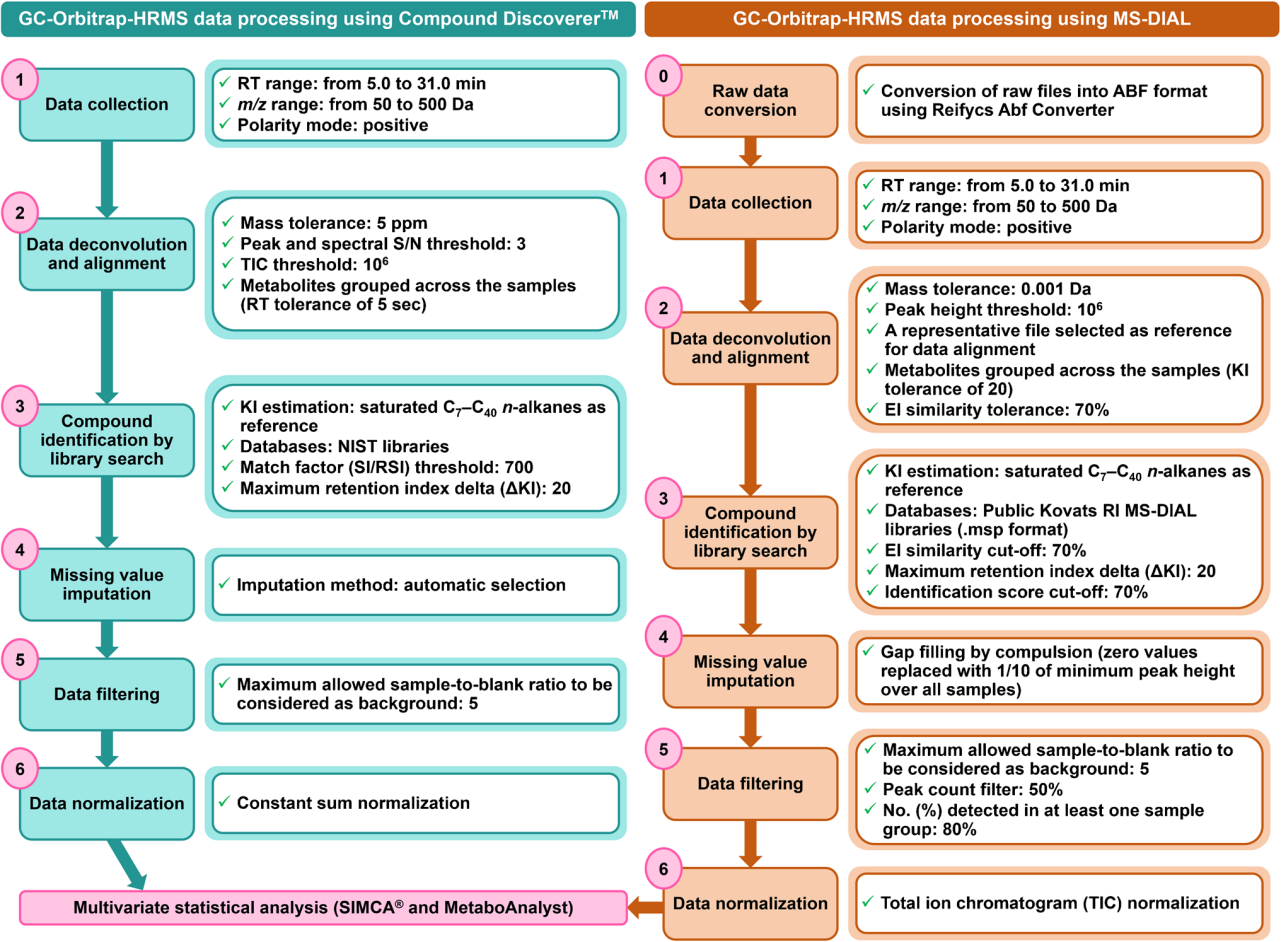
Overview of data processing workflows for the geographical differentiation of thyme using commercial Compound Discoverer™ (left) and open-source MS-DIAL software (right). Abbreviations: EI electron ionization, KI Kovats retention index, *m/z* mass-to-charge ratio, NIST National Institute of Standards and Technology, RI retention index, RT retention time, S/N signal-to-noise ratio

A customized metabolomics workflow was designed for the untargeted analysis of thyme samples using the Compound Discoverer™ software. A comparison of the data analysis workflows using Compound Discoverer™ and MS-DIAL software is shown in Fig. 1. Despite the various functionalities within each data processing software, data analysis parameters were kept as similar as possible between tested workflows to provide a comprehensive and fair comparison of current data processing methods.

First, GC-Orbitrap-HRMS raw data were imported into the platform and two sample groups were defined regarding the geographical origin factor: Poland and Spain. Additionally, procedure blanks were imported into the software for further background signal removal. For that, procedure blank files were denoted as “blanks” within the sample type categories. All comprehensive Compound Discoverer™ parameters of the designed workflow are shown in Table S1. Feature extraction was performed within the RT and *m/z* ranges of 5.0–31.0 min and 50–500 Da, respectively, selecting the positive polarity mode (corresponding to EI-MS data acquisition). Then, data deconvolution was carried out considering the following main parameters: mass tolerance of 5 ppm, spectral and peak signal-to-noise (S/N) thresholds of 3, and total ion chromatogram (TIC) threshold of 10^6^. The compounds found in the dataset were grouped across the samples using a RT tolerance of 5 s. KI estimation of deconvoluted features was achieved considering a semi-standard non-polar column (as the one used in this study) by including in the software the list of RT-number of carbons obtained for the C_7_-C_40_ *n*-alkane reference sample analyzed within the sample batch (Table S1). Then, feature identification was done by library search using the NIST libraries included in the Compound Discoverer™ software. The following library search parameters were considered for reliable compound identification: match or reverse match factor (SI/RSI) threshold of 700 and a maximum retention index delta (ΔKI) of 20 between library and experimental data (Fig. 1). Moreover, (reverse) high-resolution filtering, (R)HRF, scores were inspected as an additional parameter for accurate compound identification. For peak area refinement, missing value imputation, background filtering, and data normalization were performed. Missing value imputation was carried out by the “automatic selection” option of the software and procedure blanks were used to identify potential background features by setting in 5 the maximum allowed ratio of sample *vs*. blank to be considered as blank. The Compound Discoverer™ software offers three types of normalization: constant sum, constant median, and constant mean. In this study, data were normalized by constant sum (samples normalized to the maximum peak area sum of all samples) to avoid the potential dilution effects within the samples. The basis of this choice was providing a fair comparison between Compound Discoverer™ and MS-DIAL data processing results since constant median or constant mean normalizations were not contemplated in the MS-DIAL platform. Finally, differential analysis between sample groups was performed in the platform by considering the Poland *vs*. Spain condition, providing the corresponding *p*-values calculated by a two-tailed student’s *t*-test with Benjamini–Hochberg correction, as well as fold change analysis (FC) expressed as Log2(FC) values. Once data processing was finished, the normalized peak area data matrix was exported in .csv format for further multivariate data analysis.

#### Data processing workflow using the open-source MS-DIAL software

MS-DIAL was launched as a universal software for untargeted metabolomics analysis which is suitable for GC–MS and LC–MS data processing from different commercial platforms (Agilent Technologies, Bruker, and Thermo Fisher Scientific, among others). Moreover, MS-DIAL supports all data processing steps from raw data import to feature identification providing different public compound libraries (for EI-MS or positive/negative MS/MS data) in.msp formats. In this study, the MS-DIAL software version 4.9.221218 was used.

Regarding the data processing workflow using the MS-DIAL software, the raw data from Thermo Fisher Scientific (.raw files) were initially converted into .abf files using the Reifycs Abf Converter. Then, .abf files were imported to the MS-DIAL platform (Fig. 1). All the specific parameters used for GC-Orbitrap-HRMS data processing using the MS-DIAL software are detailed in Table S2. Briefly, during the creation of the MS-DIAL project, the following parameters were set: hard ionization (for GC–MS data), chromatography as separation type, profile for data type, and positive ion mode (for EI-MS data). Two sample groups were defined: Poland and Spain. As previously described, procedure blanks were also considered during data processing and labeled as “blank” within sample type categories. Feature extraction was performed within the *m/z* range of 50–500 Da, the RT range of 5.0–31.0 min, considering a minimum peak height of 10^6^ for Orbitrap instruments (as previously defined for the TIC threshold parameter in the Compound Discoverer™ software). Moreover, the “accurate MS” option was selected in the platform, setting 0.001 Da for the mass accuracy for centroiding and a mass slice width of 0.05 Da (suitable for accurate GC–MS data). The comparability between MS-DIAL and Compound Discoverer™ software mass accuracy criteria (0.001 Da and 5 ppm, respectively) was ensured since 0.001 Da tolerance is equivalent to 5 ppm at *m/z* 200 for Orbitrap analyzers. Peak smoothing was performed considering the following parameters: linear weighted moving average as the smoothing method (smoothing Level 2 scans and average peak width of 20 scans, according to developer recommendations). Data deconvolution was achieved using default parameters (using a sigma window value of 0.5 and an amplitude EI spectra cut-off of 10). For peak alignment considering retention indexes, the “together with alignment” option was ticked and one representative sample from the dataset was set as the reference file, setting a maximum retention index tolerance of 20 together with an EI similarity tolerance of 70%. Compound identification was performed considering retention indexes by importing into the platform the list of RT-number of carbons for the C_7_-C_40_ *n*-alkane reference file (Table S2). A retention index tolerance of 20 (as previously defined for the Compound Discoverer™-based workflow), an *m/z* tolerance of 0.5 Da (developer recommendations), an EI similarity cut-off of 70%, and an identification score cut-off of 70% were set for confident feature identification. Compound identification was done by library matching using a publicly available MS-DIAL library, i.e., “all records with Kovats RI” database (.msp format) containing EI-MS and KI data for 9062 known unique compounds (Fig. 1). Moreover, gall filling by compulsion was carried out. Finally, data filtering was done considering a peak count filter of 50% (calculated as the quotient of the number of biological replicates per group or condition and the total number of samples, i.e., 25/50). Moreover, a feature must be detected in 80% of the samples in at least one sample group to be annotated. Procedure blank files were considered to remove background features by setting in 5 the maximum allowed sample *vs*. blank fold change to be considered as blank (Fig. 1). The option “keep removable features and assign the tag” was selected for a further overview of background features identified within the dataset. Once the data processing was finished, data were normalized using the TIC option of the MS-DIAL software as the normalization factor for reliable comparison with Compound Discoverer™ constant sum normalization. The alignment result was exported as normalized peak heights into a .txt data file by setting “filtering by the ion abundance of blank sample” and “replace zero values with 1/10 of minimum peak height over all samples” options.

### Multivariate data analysis

For multivariate data analysis, commercial (SIMCA^®^ version 17 software, Umetrics, Umeå, Sweden) and open-source (MetaboAnalyst 5.0 platform available at http://www.metaboanalyst.ca) platforms were used.

Normalized peak areas (from Compound Discoverer™) and normalized peak heights (from MS-DIAL) were uploaded to SIMCA^®^ 17 or MetaboAnalyst 5.0 for statistical analysis. The common preliminary processing step was Pareto scaling. As the final aim of this authentication study was the search for Polish/Spanish thyme markers using commercial or open-source data processing tools as a case study, supervised PLS-DA modelling was employed. For that, the dataset was split into two groups: a training set consisting of 80% of the total thyme files for model-building, and a prediction set (remaining 20% of thyme samples) which was used for external model validation. An advantage of SIMCA^®^ 17 over MetaboAnalyst 5.0 is that statistical models may be externally validated by considering the correct classification rate (CCR%) obtained for the blind prediction of the samples included in the prediction set. The performance of PLS-DA models was assessed by the goodness-of-fit (R^2^Y) and the goodness-of-prediction (Q^2^) parameters. The higher the R^2^Y and Q^2^ parameters, the higher the model performance, accepting a Q^2^ cut-off > 0.5 as good predictability. PLS-DA model from SIMCA^®^ 17 was validated by internal *k*-fold cross-validation (CV) (default *k* = 7) when a CV-ANOVA *p*-value < 0.05 is obtained, by permutation tests (*n* = 100 permutations) to exclude model overfitting, and considering the CCR% achieved for the prediction set. Similarly, the PLS-DA model from the MetaboAnalyst 5.0 platform was validated by default internal 5-fold CV, which was also used to estimate the optimum number of latent variables, as well as by performing a permutation test (*n* = 100) that excludes model overfitting when an empirical *p*-value < 0.05 is achieved.

Finally, the variable importance in the projection (VIP) approach was used to determine which compounds were the most discriminant to distinguish between Polish and Spanish thyme samples. For univariate analysis of markers, Log2(FC) (FC cut-off > 1.2) and *t*-test *p*-values (*p*-values < 0.05 indicate significant differences among sample groups) were calculated using the Compound Discover™ software. However, the MS-DIAL platform did not contemplate univariate analysis and these values were mandatory obtained from the open-source MetaboAnalyst 5.0 platform.

Thus, all annotated compounds, as well as the differential ones (markers) extracted from the dataset via multivariate data analysis, were identified in good agreement with the NIST (included in the Compound Discoverer™ workflow) or the open-source EI-MS MS-DIAL library with a maximum ΔKI of 20, indicated for reliable GC-based putative identification [24]. Therefore, Level 2 of identification confidence (i.e., putatively annotated compounds or probable structures) was fulfilled for all the detected features according to established identification confidence levels using HRMS-based approaches [25, 26].

### Evaluation of commercial vs. open-source data processing performances

It is worth noting that the data processing parameters were kept as similar as possible in both tested workflows for the fairest method evaluation. However, due to the differences in functionalities of each data analysis software, this challenging comparison was not only focused on the total number of initially extracted features, since it is highly dependent on the data analysis parameters, especially those related to the feature extraction process (e.g., parameters set for the peak detection or peak alignment processes) and the further identification of extracted features (which is highly dependent on the internally available libraries). Regarding the identification of extracted features, both workflows were based on their corresponding internally available GC–MS databases, namely, NIST for Compound Discoverer™ and the “all records with Kovats RI” database (.msp format with 9062 known unique compounds) for the MS-DIAL workflow.

Therefore, for an equitable method comparison, the data processing capabilities of the two workflow strategies under study (Compound Discoverer™ and MS-DIAL software, Fig. 1) were compared in terms of the number of features extracted from each processing method, including a deeper evaluation of the number of unknown or non-library referenced, background, and duplicate features registered using each platform. Moreover, the capabilities for compound identification were assessed by comparing the final number of Level 2–identified features using available spectral libraries offered by the Compound Discoverer™ or the MS-DIAL software. Finally, reliable marker identification via SIMCA^®^ 17 or MetaboAnalyst 5.0–based multivariate data analysis was performed from Compound Discoverer™ and MS-DIAL curated data matrices, and overlap between highlighted markers was also explored as the final step of this case study.

## Results and discussion

### Results of feature extraction from untargeted GC-Orbitrap-HRMS data analysis

Representative total ion chromatograms (TICs) of Polish and Spanish thyme samples are provided in Fig. S1. TICs were characterized by a considerably high number of peaks, indicating that the compound identification process in high-complexity matrices, such as spices and herbs, may be challenging work.

**Fig. 2:**
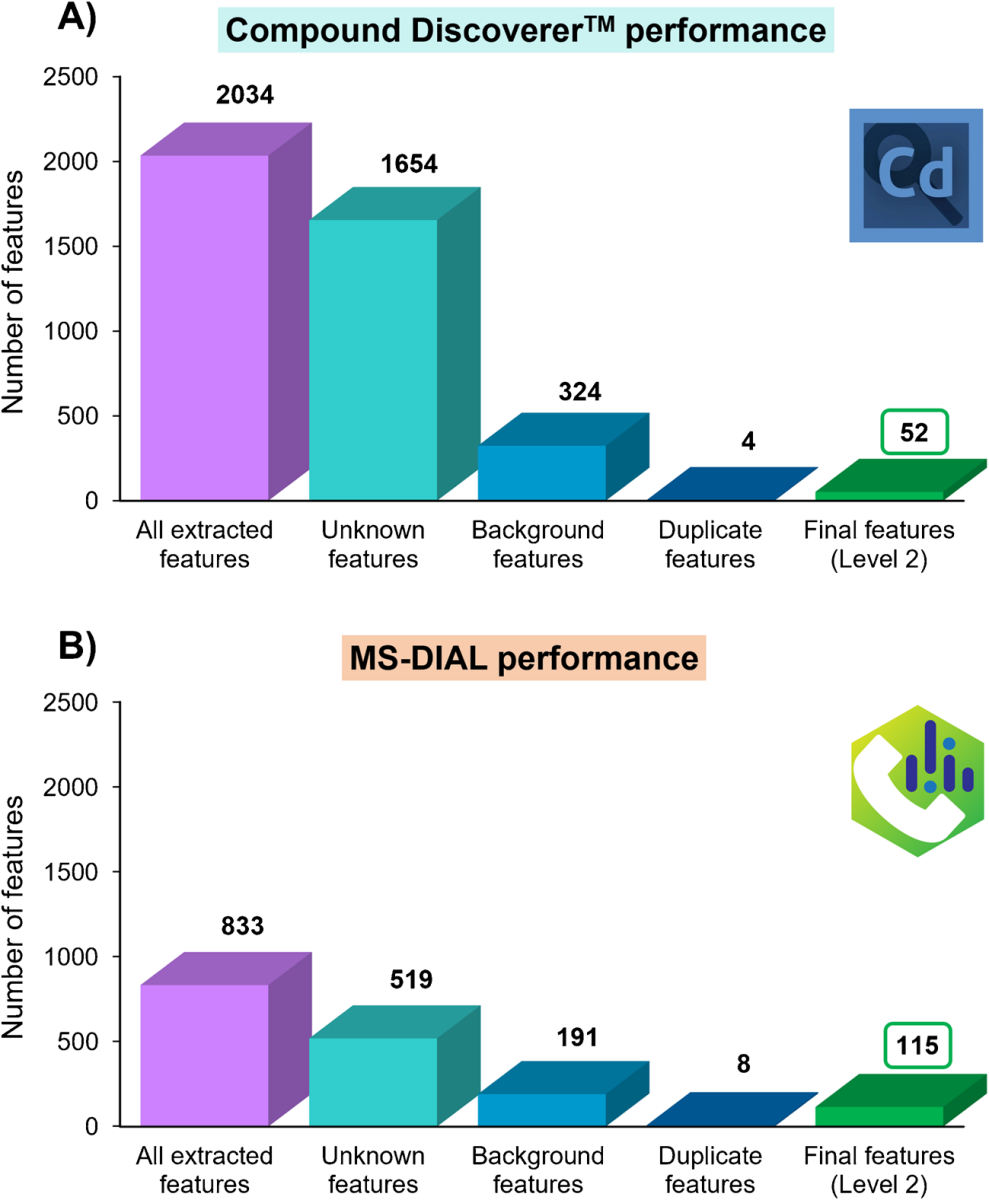
Performance comparison of data processing tools for GC-Orbitrap-HRMS untargeted metabolomics analysis using **A** Compound Discoverer™ and **B** MS-DIAL software

Compound Discoverer™ and MS-DIAL performances were comprehensively evaluated considering the following aspects (Fig. 2): (i) initial extracted features from the dataset, (ii) the number of unknown or non-annotated features, (iii) the number of background features, (iv) duplicate features within the dataset, and (v) the final number of annotated features at Level 2 of identification confidence (by library and KI matching), which were further considered for multivariate data analysis. The initially annotated features were manually curated for further multivariate data analysis for marker searching, and thus, only Level 2–identified features were kept for reliable identification of Polish *vs*. Spanish thyme marker compounds. The final number of Level 2–identified features by each different approach are included in Table S3 (Compound Discoverer™ dataset) and Table S4 (MS-DIAL dataset), and they are comprehensively described below.

#### Results of untargeted data processing using commercial Compound Discoverer™ software

For the untargeted data processing using Compound Discoverer™ workflow (Fig. 2A), a total of 2034 features were initially annotated for Polish *vs*. Spanish thyme differentiation. This high preliminary number of detected features was in good agreement with the complex sample analysis observed by qualitative inspection of thyme TICs. Most of the initial features (1654) remained unknown without library match results (Level 4 was obtained for features with unequivocal molecular formula or Level 5 if only the exact mass of interest was known [26]), with insufficient available information for their chemical annotation via NIST library matching (Fig. 2A). Since the final aim of this study was to notice the applicability of different data processing workflows for authentication studies, and thus, for the identification of thyme marker compounds, these non-library-matched features (Levels 4 and 5) were not further considered in multivariate statistical analysis. False positive feature detection should be also evaluated in data processing workflows, especially considering that background or interference compounds may be originated during the sample preparation or from instrument noise. The analysis of the procedure blanks allowed the assessment of any source of contamination introduced during any step of the measurement procedure, including from the extraction process (e.g., solvent, polypropylene, or nylon filter interferences) to the instrumental process (background features arising from mobile phases or instrument noise), as previously reported [16]. Pointing out background features by manual checking would be time-demanding bearing in mind the complexity of the matrix of study and the number of analyzed samples. In this context, the designed Compound Discoverer™ workflow allowed the automatized assessment of background features considering procedure blanks included in the analysis batch. For that, the maximum allowed ratio of sample *vs*. blank to be considered as a background compound was set at 5. As a result, the Compound Discoverer™ software marked 324 compounds as background features which were further discarded to reduce interferences and non-differential false positive features to distinguish between Polish and Spanish thyme (Fig. 2A).

Once unknown and background features were discarded, the remaining features were inspected to notice the presence of potential duplicate compounds. For duplicate identification, the remaining features were compared via “name” and corresponding InChIKey across the compound list (Table S3). It is worth noting that Compound Discoverer™ automatically provided the CAS numbers of annotated compounds (when available) retrieved from the NIST database used during compound identification. However, InChIKey should be manually consulted in the NIST library by introducing the feature CAS number. Only 4 duplicates were discarded, maintaining the feature with the highest total identification score (%) and the lowest ΔKI as the selection criteria within the same duplicates (Fig. 2A).

**Fig. 3:**
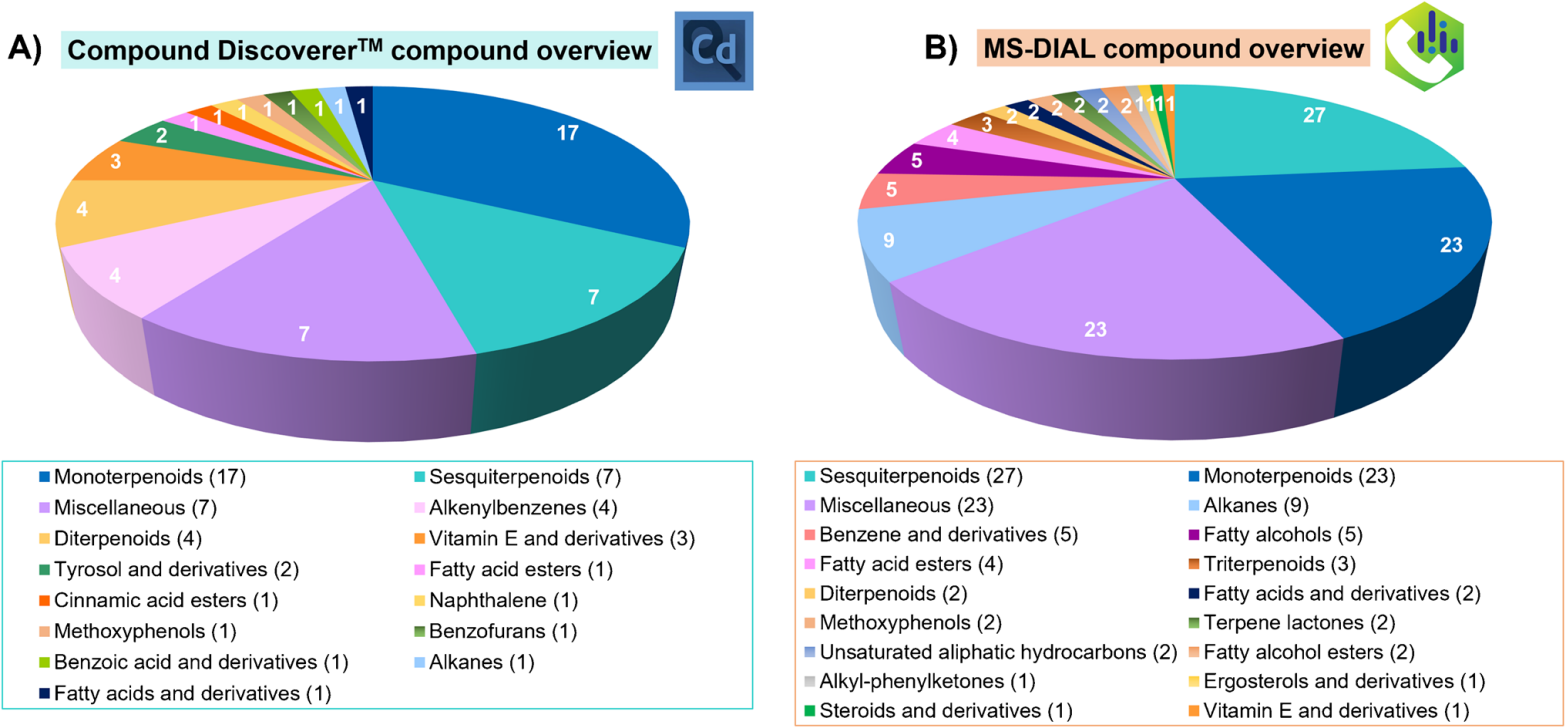
Pie charts showing the chemical classes of Level 2–annotated features used for geographical differentiation of thyme samples according to **A** Compound Discoverer™ and **B** MS-DIAL data processing results

At this point, the Compound Discoverer™ curated data matrix was manually completed by adding the compound classes of final features and their potential occurrence in herbs and spices using the Human Metabolome Database (HMDB) [27]. Thus, only 52 unique features annotated at a high confidence level (Level 2) were finally considered (Fig. 2A and Table S3), with ΔKI ≤ 20, and SI or RSI values ≥ 700. Additionally, annotated features were characterized by satisfactory HRF/RHRF scores close to 100%, and total identification scores > 90% were obtained in all the cases (Table S3). The comprehensive list of annotated compounds via Compound Discoverer™ software also included compound name, RT (min), CAS numbers, InChIKey identifiers, compound class taken from HMDB, and abundance values (expressed as constant sum normalized peak areas), among other identification parameters (Table S3). A broad compound diversity could be observed in thyme samples according to Level 2–identified features (Fig. 3A), with a clear predominance of monoterpenoid compounds (e.g., eucalyptol, sabinene hydrate, linalool) followed by sesquiterpenoids (e.g., *trans*-calamenene or epicubenol), which accounted for almost half of total Level 2–identified features. The annotation results also highlighted the presence of other miscellaneous compounds, as well as diterpenoids (e.g., ferruginol or sugiol) and alkenylbenzenes (e.g., estragole, eugenol, myristicin) in thyme extracts (Fig. 3A and Table S3). Three compounds belonging to the vitamin E and derivative group were also annotated (e.g., *δ*-tocopherol). In addition, the 10 remaining identified compounds belonged to different chemical classes, including tyrosol derivatives (e.g., tyrosol, acetate), fatty acid esters (e.g., octanoic acid, methyl ester), methoxyphenols, alkanes (e.g., heptacosane), and fatty acids and derivatives, among others (Fig. 3A and Table S3). According to the annotation results (Table S3), the Compound Discoverer™ strategy allowed the reliable identification of some metabolites, such as eugenol, 4-hydroxy-benzeneethanol, and *β*-tocopherol, in line with thyme phytochemical composition, and other detected metabolites were usually found in herbs and spices such as *α*-phellandrene, estragole, bicyclosesquiphellandrene, epicubenol, and heptacosane [27].

#### Results of untargeted data processing using open-source MS-DIAL software

As shown in Fig. 2B, the MS-DIAL data processing strategy led to a lower initial number of total features (833 extracted features) compared with the Compound Discoverer™ one (Fig. 2A). However, it would not be equitable to evaluate the processing workflow performance by the total number of initially extracted features, since it is highly dependent on the data analysis parameters. Furthermore, it should be taken into account that the open-source MS-DIAL workflow begins with the raw data conversion from .raw (GC-Orbitrap-HRMS data from Thermo Fisher Scientific) to .abf files. Indeed, this additional step makes MS-DIAL an alternative and universal processing tool for data from different instruments in comparison with commercial ones such as Compound Discoverer™, in which the workflow does not require data extension conversion but, therefore, is limited to .raw files. This conversion step might be another source of variability to explain the difference between the initial number of features extracted following both tested workflows.

Consequently, all these features should be further critically inspected by considering the annotation confidence level (if they are unknown features), background compounds, and duplicate features among all extracted metabolites for real knowledge of the data processing performance. The results in Fig. 2B also revealed that the number of library-matched features only took a small part of the total features, as previously obtained using the Compound Discoverer™–based strategy, since 519 features remained unknown after library matching using the public MS-DIAL library file. In this case, only the exact masses and EI spectrum of unknown features were provided using the MS-DIAL strategy (molecular formulas were not unequivocally assigned), achieving Level 5 of identification [26]. As previously described, unknown features were not further considered. Then, background interferences were automatically inspected by the MS-DIAL data processing workflow by considering the fold change of thyme samples *vs*. procedure blanks (set at a maximum of 5 to be considered a background feature). In this context, a lower number of background features (191) were annotated compared with the Compound Discoverer™ strategy, which was in line with the lower number of features initially extracted from the dataset (Fig. 2B). The number of duplicates was also investigated by considering the feature name or InChIKey. An advantage of the MS-DIAL software is that it directly provides different feature identifiers, including InChIKey and SMILES, as well as the compound “ontology” corresponding to the chemical class (e.g., monoterpenoids, sesquiterpenoids, fatty alcohols) (Table S4). In this case, although MS-DIAL counted with a lower number of initially extracted compounds, a slightly higher number of duplicates (8 discarded duplicate features) were highlighted in comparison with the Compound Discoverer™ data analysis. As previously indicated for the Compound Discoverer™ approach, duplicate features were compared and the one with the highest total identification score (%) and the lowest ΔKI was kept in the dataset. As a result of the manually curated MS-DIAL dataset, matching of deconvoluted EI-MS spectra to the MS-DIAL library resulted in 115 confident annotations at Level 2 (Fig. 2B and Table S4), with ΔKI ≤ 20, and total identification scores > 70%, which were further considered for multivariate data analysis and searching of thyme markers. Different factors might explain the differences observed in the final Level 2–annotated compounds within tested workflows. For instance, it is worth noting that the MS-DIAL workflow presented an additional peak area refinement filter compared to the Compound Discoverer™ one, which might contribute to the difference between the number of features extracted using both tools. As previously described, data filtering was done in both cases by removing blank features using a maximum sample-to-blank fold change of 5. Moreover, the data analysis by the MS-DIAL software was done by considering a peak count filter of 50% (calculated as the quotient of the number of biological replicates per group or condition and the total number of samples), and considering that a feature must be detected in 80% of the samples in at least one sample group to be annotated. This peak filter was not contemplated as an available customizable node in version 3.3. Compound Discoverer™ software.

Table S4 describes the 115 Level 2–identified features together with their compound names, RT (min), molecular formulas, compound classes, InChIKey identifiers, total identification scores (%), EI spectrum, and abundance values (expressed as TIC normalized peak heights), among other parameters considered during the feature identification process. An overview of compound classes of Level 2–identified features by MS-DIAL is shown in Fig. 3B. Similar to the compound overview offered by the Compound Discoverer™ software (Fig. 3A), sesquiterpenoid (e.g., *α*-cubebene or *γ*-muurolene) and monoterpenoid components (e.g., geraniol, terpinolene, fenchyl alcohol) majorly represented thyme extracts according to MS-DIAL annotation results (Table S4). The MS-DIAL strategy also revealed other 23 metabolites classified as miscellaneous compounds (e.g., 2-allyl-2-methylcyclohexanone or cumacrene) (Fig. 3B and Table S4). Tetradecane, dodecane, octane, and tetracosane were identified in thyme extracts, among other 9 alkane compounds (Fig. 3B). The MS-DIAL strategy pointed out 13 different fatty acid–related metabolites including octanoic and linolenic acids, fatty acid esters (e.g., benzylmalonic acid methyl ester and 2-methylbutyl 2-methylbutyrate), fatty alcohols (e.g., (*Z*)-9-hexadecen-1-ol), and fatty alcohol esters (e.g., citronellyl hexanoate) (Fig. 3B and Table S4). According to the results shown in Fig. 3B, diterpenoids (such as abietic acid) and triterpenoids (e.g., lanosterol and squalene) were found in thyme samples (Table S4). Other chemical classes of compounds annotated by MS-DIAL included methoxyphenols (e.g., 4-vinylguaiacol), terpene lactones (such as deoxysericealactone), and ergosterol, steroid, and vitamin E derivatives (Fig. 3B and Table S4). Some compounds such as octanoic acid, thymyl acetate, 5-isopropyl-2-methylphenol, terpinolene, *β*-ionone, and lanosterol were detected in good agreement with thyme phytochemical composition while other identified metabolites, including cedrol, hexacosane, *n*-pentadecanol, and *γ*-muurolene, are widespread among spices and herbs [27].

The comparison results from feature annotation via Compound Discoverer™ or MS-DIAL approaches (Fig. 2) revealed that, despite the apparent higher number of initial features extracted using Compound Discoverer™ software, MS-DIAL provided a higher number of final Level 2–putatively annotated compounds (52 features *vs*. 115 features). The higher number of Level 2–identified features by MS-DIAL could be explained by the different criteria and spectral libraries employed for compound identification (even though the parameters between the tested strategies were kept as similar as possible). Finally, a comparison of Level 2–identified features using Compound Discoverer™ and MS-DIAL revealed a low overlap of candidate compounds between data processing strategies (Table S3 and Table S4), as reported in other previous studies assessing the performance of data processing tools for challenging untargeted data analysis in plant metabolomics [16]. These results showed that only a small percentage of the features can be simultaneously identified by the two methods (although both workflows were designed as similar as possible). This indicates that untargeted analysis of real samples is challenging and highly dependent on the feature extraction capability of each method, including peak detection algorithms used in each case, peak filtering processing, and peak alignment (among others), resulting in differences in feature RT annotation and areas. Furthermore, additional differences may arise from the second main step in untargeted analysis, i.e., feature identification, because each workflow depends on the internal availability of GC–MS libraries.

Particularly, the corresponding Venn diagram [28] performed considering feature InChIKey (since compound name may vary depending on the data library nomenclature) highlighted only four common compounds between Compound Discoverer™ and MS-DIAL datasets, namely eucalyptol (RT = 5.63 min), sabinene hydrate (RT = 5.98 min), *cis*-2-menthenol (RT = 6.25 min), and thymohydroquinone (RT = 9.65 min) (Fig. S2). Thus, the data processing results highly encourage the evaluation not only of the initial extracted features but also the distribution of unknown, background, and duplicate features as a sign of real data processing performance.

### Multivariate data analysis results: PLS-DA supervised modeling

**Fig. 4:**
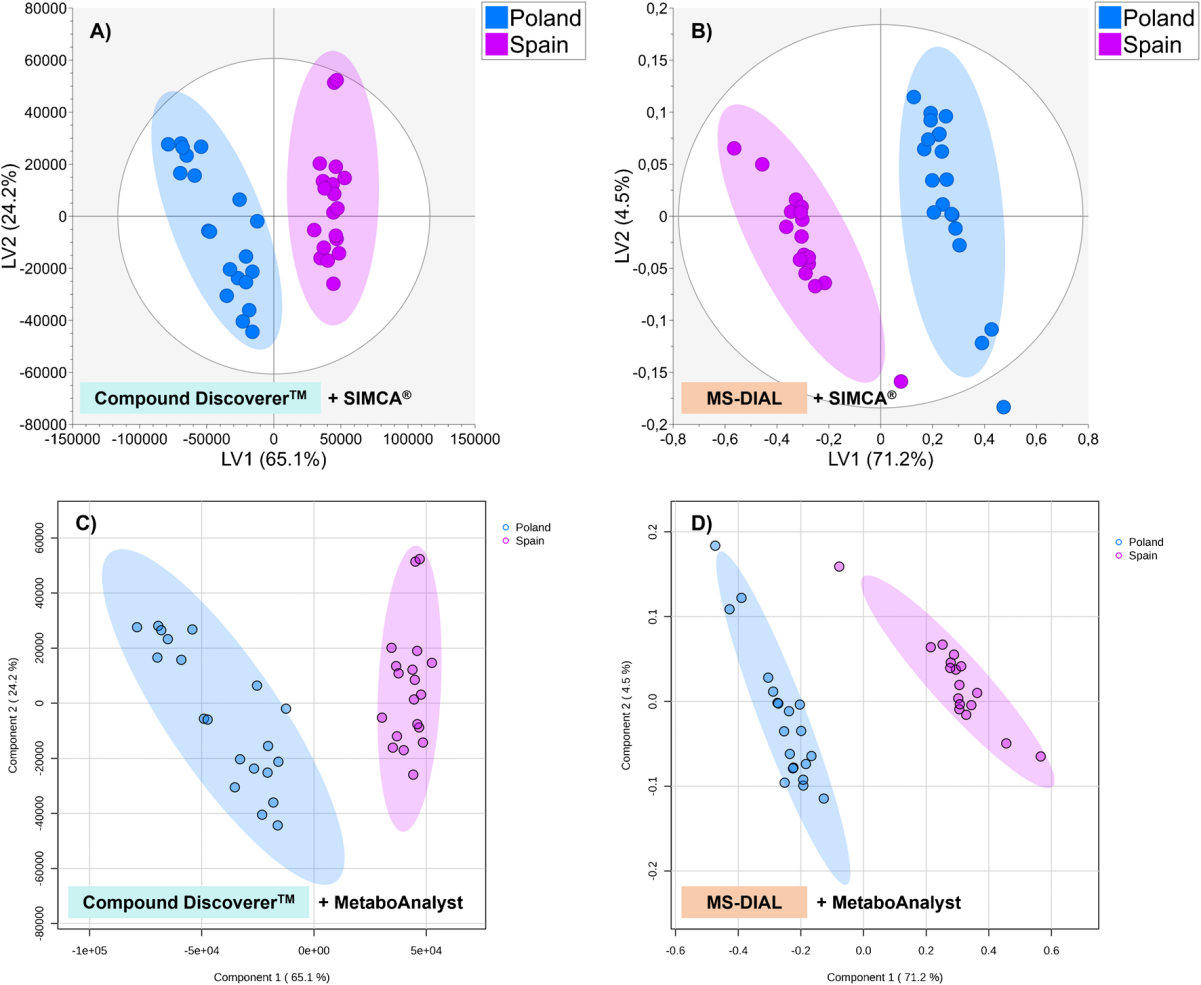
PLS-DA score plots built considering 80% of total thyme samples (training sets) showing discrimination of samples according to the geographical origin (Poland and Spain). PLS-DA score plots obtained from the SIMCA^®^ software considering **A** Compound Discoverer™ and **B** MS-DIAL datasets. PLS-DA score plots obtained from the MetaboAnalyst platform considering **C** Compound Discoverer™ and **D** MS-DIAL datasets

Supervised PLS-DA modeling built considering the training sets was used to extract information about differential compounds to discriminate within Polish and Spanish thyme. The Compound Discoverer™ data matrix further used for multivariate data analysis was made of 80% of the total observations and 52 variables (constant sum normalized peak areas of Level 2–identified features, Table S3) which was Pareto-scaled before multivariate data analysis. The PLS-DA model (built considering 80% of total observations as the training set) performed by the SIMCA^®^ software resulted in 4 latent variables (LVs) explaining 89.3% of the dataset variance considering the first two LVs (LV1 = 65.1% and LV2 = 24.2%) (Fig. 4A), with high-quality parameters (R^2^Y = 0.987, Q^2^ = 0.978, and CV-ANOVA *p*-value < 0.05, Table S5). Moreover, the permutations tests (*n* = 100 permutations) excluded model overfitting (*R*^2^, *Q*^2^ intercepts for Poland class: 0.163, − 0.400; for Spain class: 0.152, − 0.420) (Table S5). Additionally, the high model predictability indicated by the Q^2^ value was externally verified considering the maximum CCR = 100% obtained for the observations included in the prediction set. The PLS-DA score plot pointed out satisfactory sample clustering to discriminate between Polish and Spanish thyme samples along the LV1 (Fig. 4A).

Similarly, multivariate data analysis was performed considering the Pareto-scaled MS-DIAL data matrix consisting of 80% of total observations and 115 Level 2–identified features (TIC normalized peak heights, Table S4). The PLS-DA model (built considering the training set) was formed by 3 LVs, explaining 75.7% of the total variance by the first two LVs (LV1 = 71.2% and LV2 4.5%) (Fig. 4B). In this case, high model performance was also obtained (R^2^Y = 0.987, Q^2^ = 0.908, and CV-ANOVA *p*-value < 0.05, Table S5), but the MS-DIAL-based model presented slightly lower predictive ability (Q^2^ = 0.908) compared to the Compound Discoverer™-based model (Q^2^ = 0.978). Nevertheless, the high model predictability of the MS-DIAL-based model was also confirmed by the external model validation since a maximum correct classification rate (CCR = 100%) was achieved for thyme samples blindly predicted by the model (Table S5). Besides, model overfitting was discarded by permutations tests (R^2^, Q^2^ intercepts for Poland class: 0.310, − 0.274; for Spain class: 0.300, − 0.274, Table S5). The resulting PLS-DA score plot built considering the first 2 LVs showed clear discrimination between thyme samples from the two geographical locations (Poland and Spain) (Fig. 4B). MS-DIAL platform also contemplates multivariate statistical analysis (i.e., PLS-DA modeling and the VIP approach as offered by the SIMCA^®^ software to select marker compounds). Multivariate statistical analysis in MS-DIAL could be performed by choosing the following metabolites to be considered as variables (features) from the alignment result dataset: “Ref. matched” (previously defined as Level 2–identified metabolites by library matching) or “Unknown” (Level 5–identified features) tagged compounds. Nevertheless, as described during the discussion of the MS-DIAL annotation results, the MS-DIAL data matrix resulting from the alignment result needed further improvement since it contained duplicate and background features. Thus, compared to the highly curated data matrix considered for further multivariate statistical analysis by the SIMCA^®^ software, the possibility of manual selection of reliable compounds within “Ref. matched” features to be considered in statistical analysis by the MS-DIAL platform would be of interest in further software improvements.

Multivariate data analysis was also performed using the open-source MetaboAnalyst 5.0 platform. The SIMCA^®^ and MetaboAnalyst statistical results from Compound Discoverer™ and MS-DIAL data matrices were compared in terms of sample clustering and the model performance parameters (goodness-of-fit by R^2^Y and goodness-of-prediction by Q^2^). When datasets resulting from Compound Discoverer™ (Table S3) or MS-DIAL (Table S4) data processing were imported to the MetaboAnalyst platform, no normalization nor data filtering was performed since these steps were previously done during the untargeted data analysis using Compound Discoverer™/MS-DIAL software. Thus, similar to the SIMCA^®^ approach, the data matrices were only Pareto-scaled for reliable comparison between SIMCA^®^ and MetaboAnalyst statistical results. The PLS-DA model performed from the Compound Discoverer™ data matrix (52 variables) using the MetaboAnalyst platform was characterized by high-performance parameters (R^2^Y = 0.997 and Q^2^ = 0.983), explaining 89.3% of total variance considering the first 2 LVs (LV1 = 65.1% and LV2 = 24.2%), with reliable Polish *vs*. Spanish sample differentiation (Fig. 4C). As can be seen, the PLS-DA results using the SIMCA^®^ or the MetaboAnlyst approaches were highly similar (Fig. 4A and Fig. 4C), demonstrating that the platform used for multivariate data analysis was not as critical as the software used during the data processing of untargeted metabolomics data, and thus, the use of commercial or open-source statistical software depends on user’s availability. However, it is worth mentioning that the SIMCA^®^ software allows the external model validation (i.e., a prediction set may be specified and the software blindly classifies these observations into sample classes providing the result into a misclassification table), whose assessment is especially recommended in metabolomics applications focused on food authentification. This option is not currently attempted in the MetaboAnalyst 5.0 platform.

Regarding the MS-DIAL data matrix (made of 115 variables), the resulting PLS-DA model from the MetaboAnalyst platform presented R^2^Y and Q^2^ parameters of 0.996 and 0.964, respectively, explaining 75.7% of total variance considering the first 2 LVs (LV1 = 71.2% and LV2 = 4.5%), with reliable sample clustering (Fig. 4D). In this case, the MetaboAnalyst platform would notice a higher predictive ability of the model (Q^2^ = 0.964) compared with SIMCA^®^ results (Q^2^ = 0.908).

### Evaluation of significant features to differentiate Polish vs. Spanish thyme

As the final step in food metabolomics applications, once PLS-DA models were built for geographical differentiation of thyme samples, the VIP approach was used to investigate potential markers contributing to the discrimination between Polish and Spanish thyme samples. As a result, the VIP analysis identified 4 differential markers (with VIP scores > 1.00) from the Compound Discoverer™-based PLS-DA model (Fig. 4A–C), whereas a slightly higher number of differential features (5 markers) were highlighted by the VIP approach considering the MS-DIAL-based PLS-DA model (Fig. 4B–D). All VIP markers are shown in Table 1, together with their retention times (min), VIP scores (a comparison of VIP scores obtained from SIMCA^®^ software and the MetaboAnalyst platform is displayed), *p*-values (from two-samples *t*-tests performed by Compound Discoverer™ software or taken from MetaboAnalyst 5.0 platform for MS-DIAL markers), the results from fold change analysis (expressed as Log2(FC) values for Poland *vs*. Spain class comparison), and supporting literature. Moreover, further information on selected features, including ΔKI, InChIKey identifiers, and compound classes is provided in Table S6. Interestingly, most of the markers were exclusively annotated from Compound Discoverer™ or MS-DIAL approaches since only one marker (thymohydroquinone) was common between the two tested data processing strategies (Table 1).

**Table 1:** List of markers to discriminate thyme samples according to the geographical origin (Poland and Spain). Discriminant metabolites are shown with their VIP scores, *p*-values, and Log2(FC) values. Markers were selected by untargeted GC-Orbitrap-HRMS metabolomics analysis using Compound Discoverer™ or MS-DIAL data processing approaches followed by VIP analysis (VIP threshold > 1.00) of PLS-DA modeling (using SIMCA^®^ and MetaboAnalyst platforms). Supplementary data used for the putative identification (Level 2) of differential markers is shown in Table S6

No	RT (min)	Marker name (putative candidate)	Molecular formula	Exact mass (*m/z*)	VIP score		*p*-value^a^	Log2(FC)^a,b^	Occurrence in thyme species
					SIMCA^®^	MetaboAnalyst			
Highlighted by the Compound Discoverer™ approach									
1	5.55	*p*-Cymene	C_10_H_14_	134.1095	5.47	5.74	8.64 × 10^−12^	1.72	[14, 29]
2	9.65	Thymohydroquinone	C_10_H_14_O_2_	166.0993	2.87	2.82	1.93 × 10^−18^	1.33	[14, 30]
3	21.96	Vitamin E (or *α*-tocopherol)	C_29_H_50_O_2_	430.3810	1.63	1.67	4.50 × 10^−1^	0.05	First report
4	7.27	2-Methoxy-4-methyl-1-(1-methylethyl)-benzene (or thymol methyl ether)	C_11_H_16_O	164.1201	1.49	1.50	9.55 × 10^−28^	2.88	[29, 31]
Highlighted by the MS-DIAL approach									
1	8.87	3-Acetyl-2,6-dimethyl-2,5-heptadiene	C_11_H_18_O	151.0753	5.58	5.56	3.64 × 10^−12^	1.13	First report
2	9.65	Thymohydroquinone	C_10_H_14_O_2_	151.0754	2.51	2.49	7.06 × 10^−16^	1.55	[14, 30]
3	12.40	5-Methyl-1-nonyl-6,8-dioxabicyclo(3.2.1)octan-3-one	C_16_H_28_O_3_	85.0648	1.97	1.97	3.94 × 10^−15^	− 1.18	First report
4	8.81	6,9-Guaiadiene	C_15_H_24_	91.0542	1.97	1.89	2.00 × 10^−17^	1.26	First report
5	9.52	*cis*-Calamenene	C_15_H_22_	159.1168	1.14	1.16	1.39 × 10^−16^	0.81	[32]

^a^*p*-values (obtained by univariate analysis using two-sample *t*-tests) and Log2(FC) values were directly obtained from the univariate statistical analysis offered by the Compound Discoverer™ software. For the markers noticed by the MS-DIAL approach, these values were calculated by the MetaboAnalyst 5.0 platform

^b^Results of fold change (FC) analysis performed for the comparison of Polish *vs*. Spanish thyme samples expressed as Log2(FC) values

#### Thyme markers revealed by the Compound Discoverer™–based approach

Four markers were highlighted for thyme geographical differentiation using the Compound Discoverer™ dataset, namely *p*-cymene, thymohydroquinone, vitamin E (or *α*-tocopherol), and 2-methoxy-4-methyl-1-(1-methylethyl)-benzene (or thymol methyl ether) (Table 1). The results revealed that most of the markers belong to the monoterpenoid family (Table S6).

The PLS-DA model followed by the VIP approach revealed the feature with RT = 5.55 min (VIP scores of 5.47–5.74) as the most differential one to discriminate between Polish and Spanish thyme (Table 1). This feature was putatively identified as *p*-cymene (ΔKI = 7 with a total identification score of 97.2%, Table S3) and it was found significantly up-accumulated in Polish thyme (Log2(FC) value of 1.72 and *p*-value of 8.64 × 10^−12^) (Table 1). In line with these results, *p*-cymene was described in previous literature as a major constituent of commercial *Thymus vulgaris* L. [14, 29].

**Fig. 5:**
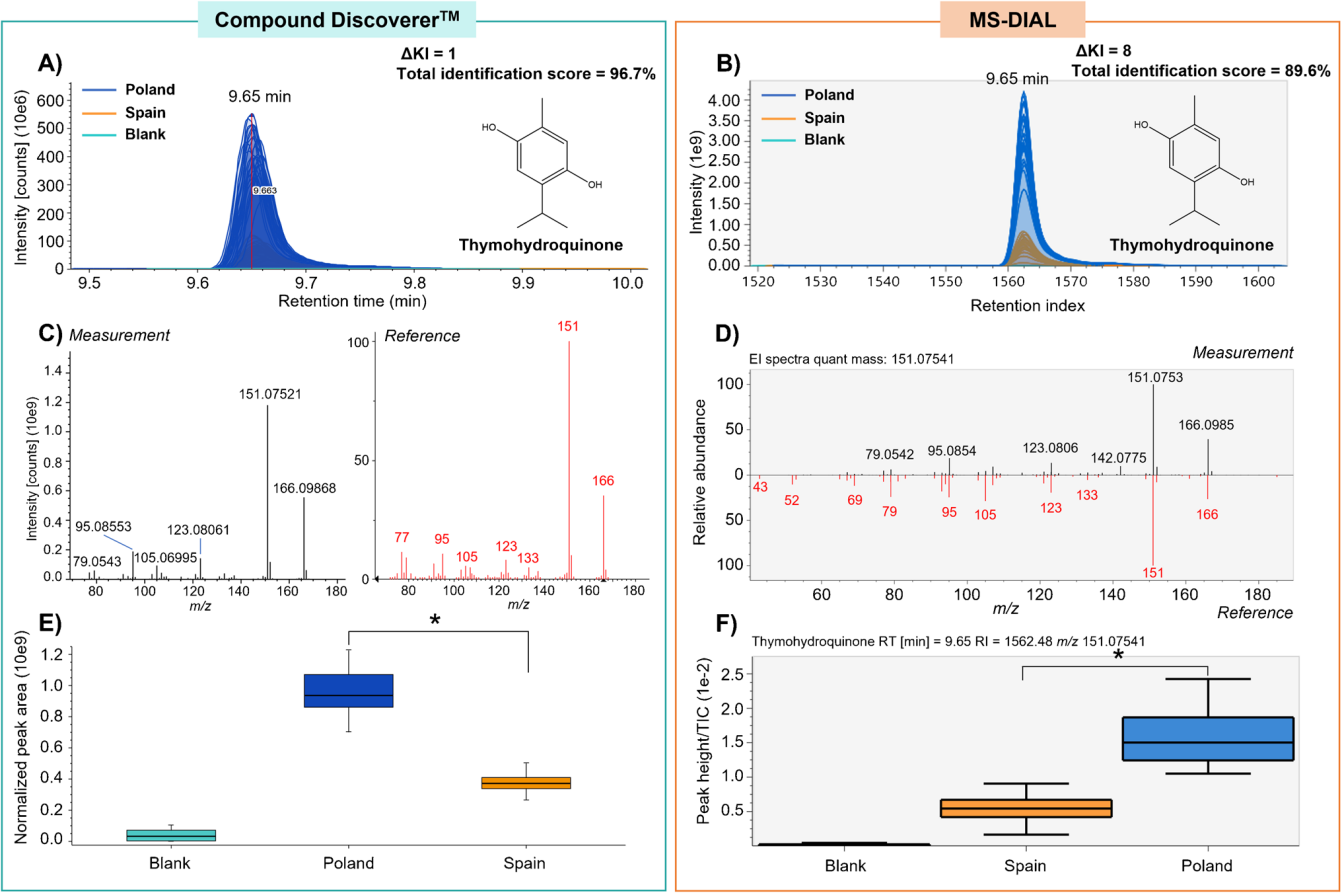
Overview of compound identification workflow by Compound Discoverer™ (left) and MS-DIAL software (right) showing the Level 2–identification of thymohydroquinone in thyme as an example. **A**, **B** Aligned chromatograms showing feature peaks at RT = 9.65 min detected within the thyme samples. **C**, **D** EI-MS spectrum of thymohydroquinone in thyme samples (measurement in black) compared to the reference spectrum from the NIST or public MS-DIAL library (reference in red). **E**, **F** Boxplot analysis displaying significant differences in relative levels of thymohydroquinone between Polish and Spanish thyme (significant differences are indicated by an asterisk using a two-sample *t*-test, *p*-value < 0.05)

Thymohydroquinone (RT of 9.65 min, ΔKI = 1, and annotated with a total identification score of 96.7%), a monoterpenoid reported within the phytochemical composition of thyme [14, 30], was highlighted as a differential compound (VIP scores of 2.82–2.87) to discriminate Polish from Spanish thyme (Table 1). Fold change analysis (Log2(FC) = 1.33) and the two-sample *t*-test results (*p*-value of 1.93 × 10^−18^) showed that thymohydroquinone may be used as a marker of thyme cultivated in Poland since this metabolite was found up-accumulated compared to Spanish thyme extracts (Table 1). Figure 5 describes a comparison of the Compound Discoverer™ and MS-DIAL workflow for the identification of thyme markers, showing the annotation overview of thymohydroquinone as an example. Firstly, all chromatograms of the feature were properly aligned by the RT or the KI (Fig. 5A–B). Then, putative annotation was performed by comparing the acquired mass spectra with the NIST or the MS-DIAL libraries (Fig. 5C–D), obtaining in both cases high total identification scores (> 85%). Additionally, an acceptable difference between the experimental KI and the library KI (ΔKI < 20) was obtained using both platforms. Finally, box plots revealed significant enrichment of thymoquinone in Polish thyme compared to Spanish one (*p*-value < 0.05) (Fig. 5E–F).

Vitamin E (RT of 21.96 min, ΔKI = 16, and associated with a total identification score of 97.7%), with VIP scores of 1.63–1.67, was reported for the first time as a potential geographical marker of thyme due to the enhanced content in Polish thyme compared with lower concentrations found in Spanish samples (Log2(FC) of 1.67), but in this case, despite being highlighted as a high-contribution compound for Polish *vs*. Spanish thyme differentiation by the PLS-DA and the VIP analysis, these content differences were not significant (*p*-value > 0.05) according to the *t*-test findings (Table 1). Vitamin E (so-called *α*-tocopherol) has been identified in other related matrices such as saffron [33]. Furthermore, an *α*-tocopherol derivative (i.e., *α*-tocopherol succinate) was recently revealed as a potential marker of thyme submitted to post-harvest sterilization practices [8].

The remaining metabolite (RT = 7.27 min and VIP scores of 1.49–1.50) was Level 2–identified as thymol methyl ether (ΔKI = 1 with a total identification score of 97.4%) (Table 1 and Table S3). Thymol methyl ether was also found at significantly higher relative concentrations in Polish thyme (Log2(FC) of 2.88 and *p*-value of 9.55 × 10^−28^) (Table 1). Although the occurrence of thymol methyl ether was reported in common thyme (*Thymus vulgaris* L.) [29] and other thyme species [31], this study highlighted for the first time its novelty as a thyme marker for Polish origin discrimination.

Overall, the univariate data analysis performed for markers annotated via Compound Discoverer™ software and further selected using the VIP approach revealed the following outcome: all differential metabolites (mainly belonging to the monoterpenoid class) may be used as markers of the Polish cultivation area as a result of their enrichment compared to thyme produced in Spain. In line with these findings, previous thyme studies revealed that Polish thyme was mainly characterized by monoterpenoids, representing almost 90% of its total volatile phytochemical composition [34].

#### Thyme markers revealed by the MS-DIAL-based approach

Five discriminant metabolites were extracted from the MS-DIAL dataset to differentiate between Polish and Spanish thyme samples (Table 1). Compared with Compound Discoverer™ markers, a slightly larger variety of compound classes was found within the MS-DIAL differential compounds, mainly represented by monoterpenoids, followed by sesquiterpenoids, and other miscellaneous compounds (Table S6).

In detail, most of the annotated marker metabolites were monoterpenoids, namely 3-acetyl-2,6-dimethyl-2,5-heptadiene and thymohydroquinone (Table 1). In fact, 3-acetyl-2,6-dimethyl-2,5-heptadiene (VIP scores of 5.58–5.56) and thymohydroquinone (VIP scores of 2.49–2.51) were the most discriminant variables according to the VIP findings (associated with the highest VIP scores) (Table 1). Particularly, the occurrence of thymohydroquinone is reported in common thyme [14, 30], whereas 3-acetyl-2,6-dimethyl-2,5-heptadiene occurrence in thyme was firstly reported by this study, revealing its novelty as a thyme marker for Polish origin discrimination (Table 1). Considering the fold change results, all the monoterpenoid markers were significantly (*p*-values < 0.05) up-accumulated in Polish thyme (positive Log2(FC) values between 1.13 and 1.55 were obtained) (Table 1).

Two markers belonging to the sesquiterpenoid family were annotated, namely 6,9-guaiadiene and *cis*-calamenene (Table 1). 6,9-guaiadiene (RT = 8.81 min and VIP scores 1.89–1.97) was Level 2–identified by considering a ΔKI = 12 and a total identification score of 86.5% (Table S4 and Table S6). This compound has not been described in thyme but it is found in other condiments such as ginger essential oil (*Z. officinale* L.) [35]. *cis*-calamenene (RT = 9.52 min, VIP scores 1.14–1.16, ΔKI = 10, and total identification score of 87.9%) (Table S4 and Table S6) was found in line with previous literature on thyme phytochemical composition [32] (Table 1). The inspection of fold change results obtained for sesquiterpenoid markers revealed the same up-accumulation trend in Polish thyme (Log2(FC) values ranging from 0.81 to 1.26) (Table 1).

The remaining marker was identified as a miscellaneous compound: 5-methyl-1-nonyl-6,8-dioxabicyclo(3.2.1)octan-3-one (Table 1). This metabolite was putatively annotated for the first time in this GC-Orbitrap-HRMS study. Interestingly, 5-methyl-1-nonyl-6,8-dioxabicyclo(3.2.1)octan-3-one (VIP score of 1.97) was found at significantly higher levels in Spanish thyme extracts compared to Polish ones (Log2(FC) of − 1.18) (Table 1), and thus, it was revealed as a marker of Spanish production area.

Thus, compared to the Compound Discoverer™ approach possibilities, the MS-DIAL data processing strategy revealed not only markers found significantly up-accumulated in thyme produced in Poland but also key metabolites to distinguish the Spanish cultivation area.

## Conclusions

In the present study, two different GC-Orbitrap-HRMS data processing approaches (Compound Discoverer™ and MS-DIAL) were comprehensively evaluated for their use in plant-derived food authenticity applications by considering the Polish *vs*. Spanish thyme marker searching as a case study.

In terms of compound detection, the simultaneous comparison of both data processing strategies has revealed that resulting raw data matrices should be properly curated considering the background, unknown, and potential duplicate features before performing multivariate data analysis to extract differential metabolites, being a time-consuming but essential step for unbiased results. In fact, among the total 2034 (Compound Discoverer™) or 833 (MS-DIAL) extracted features, only 52 or 115 ones, respectively, were Level 2–annotated and further considered, leaving the structures of most detected features as unknown (corresponding to Level 4 or Level 5 of identification confidence), and thus, representing one of the main limitations of current data processing options for untargeted data analysis. In detail, only four Level 2–identified features were common between both datasets, demonstrating that the selection of the library plays a crucial role in determining the compound overview in thyme samples, despite both approaches used GC–MS-based libraries of standardized EI-MS spectrum with KI data (at 70 eV). Although most of the annotated features were exclusive between datasets, the compound class overview did not display disparate outcomes among them, since annotated metabolites in thyme samples were mainly represented by monoterpenoids, and sesquiterpenoids, among other miscellaneous compounds. The further statistical results showed that PLS-DA with the VIP analysis was a powerful tool for the reliable putative identification of 4 and 5 thyme markers by Compound Discoverer™ and MS-DIAL platforms, respectively. Similarly, most of the annotated markers were exclusive from each tested platform. The univariate statistical data analysis findings revealed the enrichment of most of the marker metabolites in Polish thyme, such as *p*-cymene, thymohydroquinone, and *cis*-calamenene. Interestingly, the MS-DIAL platform was able to extract metabolites distinctive of Spanish thyme (i.e., 5-methyl-1-nonyl-6,8-dioxabicyclo(3.2.1)octan-3-one).

Therefore, this study demonstrates that the MS-DIAL platform is a promising open science tool that can feasibly applied in food authenticity issues focused on the search for differential marker compounds, although it presents some limitations (e.g., univariate data analysis, including fold change analysis or basic statistics as *t*-tests, would be of great interest in comparative analysis of markers). Compound Discoverer™ remains a reliable data processing tool with highly customizable workflows, including univariate data analysis for marker interpretation and class comparison. Overall, this study has revealed that performing a fair evaluation of annotated features in untargeted analysis of complex matrices such as spices and herbs is especially challenging. Nevertheless, the findings encourage the use of both approaches for reliable identification of markers depending on the user’s availability. In this sense, this study provides a guidance of Compound Discoverer™/MS-DIAL workflows for users who worked in plant metabolomics based on GC-HRMS data.

### Supplementary Information

Below is the link to the electronic supplementary material.

Supplementary file1 (PDF 438 KB)

Supplementary file2 (XLSX 352 KB)

## Data Availability

All datasets generated or analyzed during this study are included in this published article and its supplementary information files. Open access at the following Mendeley repository: 10.17632/969xws24rm.2.
