# Supplementary material for: Comparison of data processing strategies using commercial vs. open-source software in GC-Orbitrap-HRMS untargeted metabolomics analysis for food authentication: thyme geographical differentiation and marker identification as a case study
Source: Anal Bioanal Chem. 2024 May 28;416(18):4039–55. doi: 10.1007/s00216-024-05347-0 (PMC11249438; doi:10.1007/s00216-024-05347-0)
Supplement: Supplementary file 1 — Supplementary file1 (PDF 438 KB) [file 216_2024_5347_MOESM1_ESM.pdf]

Journal name: Analytical and Bioanalytical Chemistry

SUPPLEMENTARY INFORMATION to the paper entitled: Comparison of data processing strategies using commercial *vs.* open source software in GC-Orbitrap-HRMS untargeted metabolomics analysis for food authentication. Thyme geographical differentiation and marker identification as a case study

Araceli Rivera-Pérez*, Antonia Garrido Frenich

Research Group "Analytical Chemistry of Contaminants", Department of Chemistry and Physics, Research Centre for Mediterranean Intensive Agrosystems and Agrifood Biotechnology (CIAIMBITAL), Agrifood Campus of International Excellence (ceiA3), University of Almeria, E-04120, Almeria, Spain

* Corresponding author.

E-mail address: arp800@ual.es (A. Rivera-Pérez).

ORCID CODES AND E-MAIL ADDRESS

Araceli Rivera-Pérez: 0000-0003-1099-7185 (arp800@ual.es)

Antonia Garrido Frenich: 0000-0002-7904-7842 (agarrido@ual.es)

Table of contents

Supplementary tables

Table S1. Processing parameters used for GC-Orbitrap-HRMS data analysis using the Compound Discoverer software (see attached excel file).

Table S2. Processing parameters used for GC-Orbitrap-HRMS data analysis using the MS-DIAL software (see attached excel file).

Table S3. List of detected features (Level 2-annotated) in thyme samples by untargeted GC-Orbitrap-HRMS analysis using the Compound Discoverer software (see attached excel file).

Table S4. List of detected features (Level 2-annotated) in thyme samples by untargeted GC-Orbitrap-HRMS analysis using the MS-DIAL software (see attached excel file).

Table S5. Performance and validation parameters for supervised PLS-DA models built for geographical differentiation of thyme using SIMCA and MetaboAnalyst statistical tools (see attached excel file).

Table S6. Additional information of selected marker metabolites from Compound Discoverer and MS-DIAL datasets.

Supplementary figures

Fig. S1. Representative total ion chromatograms (TICs) of thyme from (A) Spain and (B) Poland.

Fig. S2. Venn diagram showing the common identified features between Compound Discoverer and MS-DIAL datasets. Common metabolites are highlighted.

Table S6. Additional information of selected marker metabolites from Compound Discoverer and MS-DIAL datasets.

No.	RT (min)	Marker name	Compound class[a]	InChIKey[b]	ΔKI[c]
		**Highlighted by the Compound Discoverer™ approach**			
**1**	5.55	*p*-Cymene	Monoterpenoid	HFPZCAJZSCWRBC-UHFFFAOYSA-N	7
**2**	9.65	Thymohydroquinone	Monoterpenoid	OQIOHYHRGZNZCW-UHFFFAOYSA-N	1
**3**	21.96	Vitamin E (or α-tocopherol)	Vitamin E and derivatives	GVJHHUAWPYXKBD-IEOSBIPESA-N	16
**4**	7.27	2-Methoxy-4-methyl-1-(1-methylethyl)-benzene (or thymol methyl ether)	Monoterpenoid	LSQXNMXDFRRDSJ-UHFFFAOYSA-N	1
**No.**	**RT (min)**	**Marker name**	**Compound class[a]**	**InChIKey[b]**	**ΔKI[c]**
		**Highlighted by the MS-DIAL approach**			
**1**	8.87	3-Acetyl-2,6-dimethyl-2,5-heptadiene	Monoterpenoid	XUOWQIBGRJIEDN-UHFFFAOYSA-N	12
**2**	9.65	Thymohydroquinone	Monoterpenoid	OQIOHYHRGZNZCW-UHFFFAOYSA-N	8
**3**	12.40	5-Methyl-1-nonyl-6,8-dioxabicyclo(3.2.1)octan-3-one	Miscellaneous	PIMWQQQJSCEAQD-UHFFFAOYNA-N	13
**4**	8.81	6,9-Guaiadiene	Sesquiterpenoid	IFJYSOWCCJPUJS-UHFFFAOYSA-N	12
**5**	9.52	*cis*-Calamenene	Sesquiterpenoid	PGTJIOWQJWHTJJ-UHFFFAOYSA-N	10

[a] Compound class retrieved from the Human Metabolome Database (HMDB). D.S. Wishart et al., HMDB 5.0: the Human Metabolome Database for 2022, *Nucleic Acids Res*. 50 (2022) D622–D631. https://doi.org/10.1093/nar/gkab1062.
[b] InChIKey identifiers retrieved from the NIST library (for markers highlighted by the Compound Discoverer™ approach) or directly provided by the MS-DIAL software.
[c] ΔKI: Difference between experimental KI and theoretical KI (Kovats index).

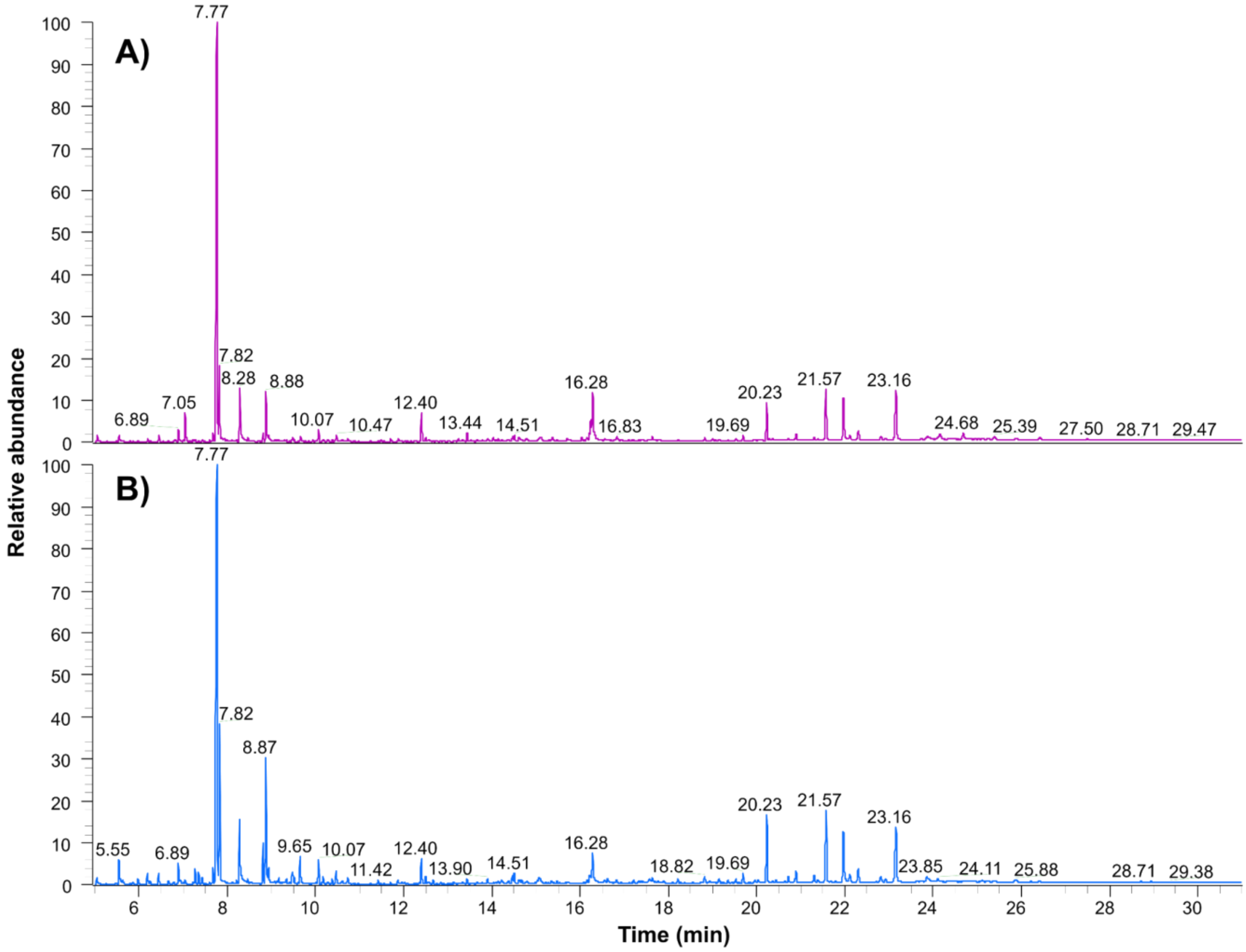

Fig. S1. Representative total ion chromatograms (TICs) of thyme from (A) Spain and (B) Poland.

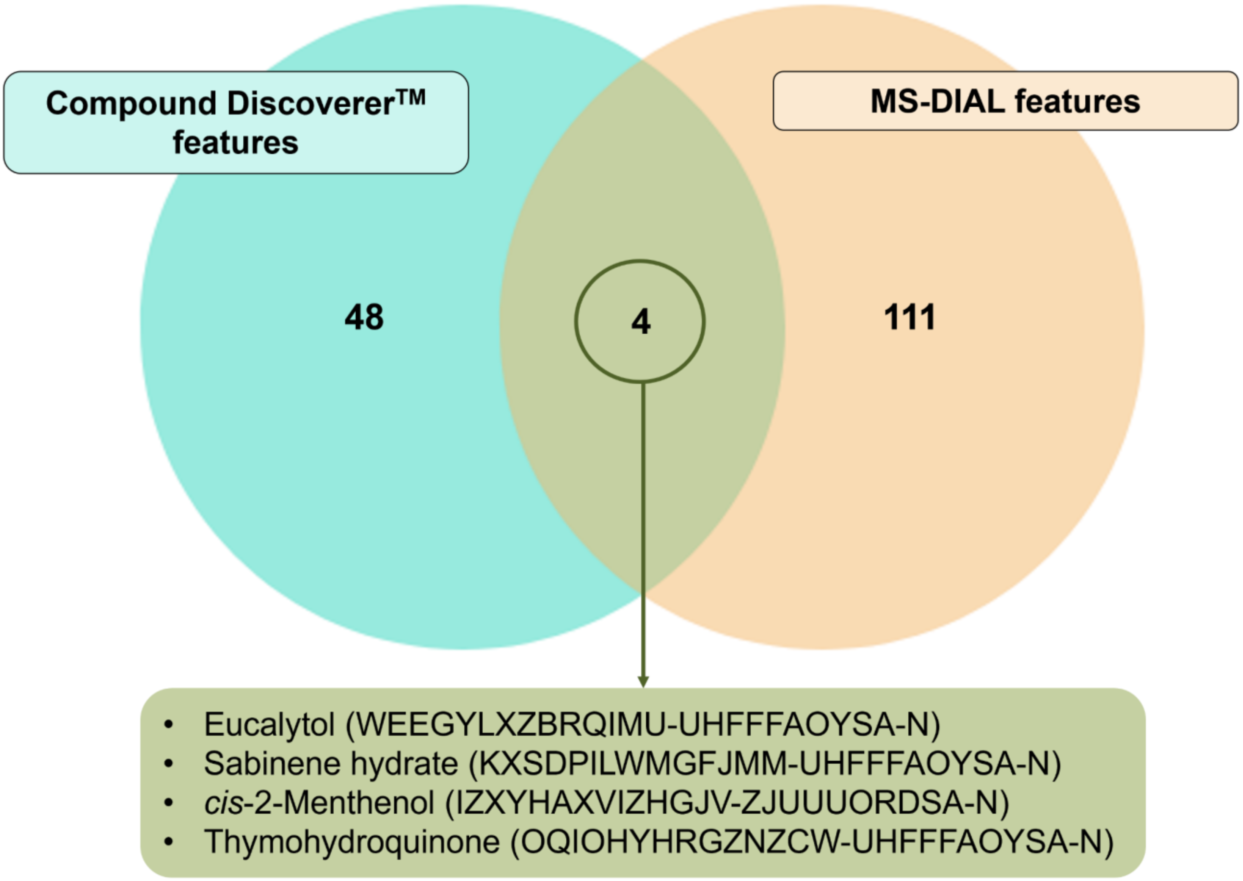

Fig. S2. Venn diagram showing the common identified features between Compound Discoverer and MS-DIAL datasets. Common metabolites are highlighted.
